# Immediate and Maintained Effects of Explicit and Contextualized Narrative and Expository Language Intervention for Children with Developmental Language Disorder

**DOI:** 10.3390/children13040496

**Published:** 2026-04-01

**Authors:** Douglas B. Petersen, Giana H. Hunsaker, Taylor Magleby, Jessica Waldron

**Affiliations:** 1 Department of Communication Sciences and Disorders, Baylor University, One Bear Place #97332, Waco, TX 76798, USA; jessica_waldron1@baylor.edu; 2 Little Language Co., 102 Dunbar Ave, Beckley, WV 25801, USA; giana.hunsaker.slp@gmail.com; 3 Nebo School District, Spanish Fork, UT 84660, USA; taylor.magleby@gmail.com

**Keywords:** narrative, expository, language intervention, structured literacy, oral language, reading comprehension

## Abstract

**Highlights:**

**What are the main findings?**

- A brief, small-group oral language intervention improved narrative language skills in young children with DLD, with gains maintained over time.
- The intervention produced a positive but nonsignificant trend on expository language outcomes.

**What are the implications of the main finding?**

- Teaching both narrative and expository language can strengthen early academic language development.
- Brief, structured small-group instruction can be used in schools to support children at risk for language and literacy difficulties.

**Abstract:**

Background/Objectives: Children develop the ability to construct meaning from language long before they learn to read. These foundational language processes support learning across academic contexts and form the basis for later reading and writing. However, many students, particularly those with Developmental Language Disorder (DLD), enter school with weaknesses in oral academic language that limit their ability to understand and express increasingly complex classroom discourse. Despite the central role of narrative and expository language in early instruction, explicit and systematic intervention targeting both discourse genres remains uncommon in the early grades. The purpose of this randomized controlled trial was to examine the immediate and maintained effects of a structured, small-group oral language intervention targeting both narrative and expository discourse for early elementary-age students with and without DLD. Methods: Participants (*N* = 80) were kindergarten through second-grade students identified as having DLD or significantly weak narrative language performance and were randomly assigned to an intervention condition or business-as-usual control. Narrative outcomes were collected for all participants at pretest, posttest, and a two-month follow-up, and expository outcomes were collected at posttest. Results: Results indicated statistically significant intervention effects for narrative language at posttest, with gains maintained at follow-up. Treatment effects were not moderated by language status, and subgroup analyses demonstrated large effects for students with DLD. Regression analyses indicated a non-significant intervention effect on expository outcomes. Conclusions: Findings provide experimental evidence that explicit, contextualized narrative and expository language instruction delivered in brief small-group sessions can produce meaningful and durable improvements in narrative language for young children, including those with DLD.

## 1. Introduction

Children develop the ability to construct meaning from language long before they learn to read [1]. Through everyday experiences with stories, explanations, and conversation, they learn to organize events, integrate vocabulary, track causal relations, and draw inferences from spoken discourse. These foundational language processes support learning across academic contexts and form the basis for later reading and writing. Although skilled reading requires both word recognition and language comprehension [2], a substantial body of evidence indicates that comprehension difficulties often reflect weaknesses in oral academic language rather than in decoding alone [3,4]. As children move beyond the earliest stages of literacy acquisition, decoding and language comprehension increasingly rely on distinct cognitive processes, with comprehension requiring the construction of coherent mental representations from linguistic input [5].

Recognizing the central role of language, contemporary academic standards emphasize students’ ability to understand and produce complex discourse, including narrative and expository language, from the early elementary grades [6]. Narrative language refers to the ability to comprehend and produce structured accounts of events, typically organized around characters, an initiating event, and a resolution, the kind of discourse encountered in stories, personal recounts, and fictional texts. Expository language, by contrast, involves the comprehension and production of informational discourse organized around topics, main ideas, and supporting details, the kind of language found in science and social studies texts, explanations, and academic lectures. However, instructional practice in many classrooms continues to prioritize word-level skills, while explicit, systematic instruction in oral academic language remains limited [7,8]. For children with Developmental Language Disorder (DLD), which is a neurodevelopmental condition characterized by significant and persistent difficulties in understanding and producing spoken language in the absence of known biomedical causes [9], this imbalance is especially consequential. Without targeted support for narrative and expository language, early weaknesses in oral comprehension may persist, constraining children’s ability to engage with academic content across modalities [10]. Early intervention that directly targets oral academic language is therefore a critical opportunity to support learning and participation during the foundational years of schooling.

## 2. The Relationship Between Reading Comprehension and Oral Language

Oral academic language is foundational for reading comprehension and overall academic success [11,12,13]. Unlike the informal language of everyday conversation, oral academic language reflects the linguistic demands of schooling and written text, including less frequent vocabulary, more abstract meanings, and increasingly complex syntax [14]. Longitudinal evidence underscores the importance of these early language skills. Longitudinal evidence underscores the importance of early oral language skills for later reading comprehension: Lervåg et al. [15] demonstrated that vocabulary, grammar, and inferencing in early childhood made strong and independent contributions to reading comprehension across the school years, with oral language remaining a significant predictor even after accounting for decoding ability. A growing body of research highlights oral language as a particularly critical factor for academic success, especially for children at risk for language-based learning difficulties, including culturally and linguistically diverse students [12,16]. Children with DLD, in particular, demonstrate significant weaknesses in narrative language performance relative to their typically developing peers, with deficits that are robust across age groups and discourse contexts [17].

Understanding why children with DLD are particularly vulnerable requires examining the close relationship between oral language and reading comprehension, which reflects shared underlying cognitive and linguistic processes. Comprehension of spoken and written language requires integrating vocabulary knowledge, syntax, inferencing, and verbal working memory to construct coherent mental representations of text [18,19]. Vocabulary acquired through oral language transfers from speaking to reading and writing. Additionally, the ability to draw inferences from spoken texts is critical to developing listening and reading comprehension. A firm syntactic foundation is also vital for further expanding an individual’s lexicon and facilitating listening and reading comprehension. Longitudinal evidence indicates that the effects of vocabulary, grammar, working memory, and inferencing on reading comprehension closely mirror their effects on listening comprehension, underscoring the shared linguistic foundations of both modalities [15]. Kieffer and Vukovic [19] explored heterogeneity in the sources of reading difficulties among children who are minority-language learners and native English speakers. They found that in their entire sample of first- through third-grade students, 80% with low reading comprehension demonstrated weaknesses in language comprehension alone.

At the same time, listening comprehension differs from reading comprehension in that it is not constrained by the cognitive demands of decoding [18]. This distinction helps explain why weaknesses in oral language may be evident well before formal reading instruction begins and why many children later identified with reading comprehension difficulties show early deficits in oral language, particularly with narrative and expository language. For children with DLD, these early weaknesses may be particularly persistent, placing them at heightened risk for ongoing difficulties in comprehending and producing academic language across spoken and written contexts [20].

Central to this relationship are two discourse forms—narrative and expository language—both of which are explicitly embedded in early elementary curricula and both place demands on children that, for those with DLD in particular, have not been met through typical classroom instruction alone [6,21].

## 3. Narrative Language: Development and Intervention

Narrative language is one of the primary forms through which children develop and demonstrate oral academic language competence, and it represents a critical site for early intervention [22]. Early narrative skills typically emerge through immersion in narrative-rich environments, including conversation, oral storytelling, shared book reading, and media exposure [23,24,25,26]. For many children, these everyday language experiences are sufficient to support the development of foundational narrative skills. However, children with limited exposure to rich conversational discourse or those with underlying language learning difficulties often demonstrate delays in narrative development that do not resolve without explicit instruction [27,28]. For these children, narrative language does not develop incidentally; it requires systematic support.

A growing body of research demonstrates that explicit, systematic narrative language intervention can substantially improve oral language outcomes, particularly when targeting story grammar and language complexity. Two key components of cohesive narrative production are story grammar and language complexity [29]. Story grammar comprises the structural elements that organize events into a coherent episode, including a character, setting, problem, internal response, plan, attempt, consequence, and resolution [30]. Language complexity encompasses the syntactic and lexical features that enable speakers to express relationships among events, such as temporal and causal subordinate clauses. Explicit instruction targeting both story grammar and language complexity is necessary for many children, particularly those with language learning difficulties. Effective instruction requires frequent opportunities to respond, systematic scaffolding and fading of visual supports, immediate corrective feedback, and graduated prompting to support independence [29]. Such instruction has been shown to be functional, efficient, and broadly relevant for improving narrative language outcomes [31].

A systematic review by Pico et al. [32] found that effective characteristics of narrative language intervention consistently included explicit teaching of story grammar elements, visual supports, verbal scaffolding, and repeated opportunities for narrative production, with moderate to large effects on both narrative comprehension and production across diverse learner populations. Because narrative retelling requires children to explicitly recall and organize linguistic elements, instruction that systematically teaches story grammar enables students to internalize narrative structure and apply it to their own productions.

For children with DLD specifically, who are less likely to develop narrative competence through incidental exposure alone, this kind of explicit instruction is not supplementary; it is essential. Gillam and Gillam [28] demonstrated that school-age children receiving narrative-based language intervention showed moderate to large gains in narrative proficiency and vocabulary. Importantly, these gains extended beyond narrative structure itself: students showed spontaneous increases in syntactic complexity, suggesting that exposure to and production of well-formed narrative models can support broader language development even without direct instruction in syntax.

Narrative language intervention has also been shown to be effective when embedded within multi-tiered systems of support. Spencer et al. [33] implemented a multi-tiered narrative intervention in a Head Start preschool serving a diverse student population and reported significant improvements in narrative retell performance. As students’ narrative skills improved, they also produced longer and more linguistically complex utterances. These findings align with prior research demonstrating narrative language instruction delivered across tiers can support language development for both typically developing children and those with language difficulties [27,34,35]. Several studies have examined narrative language intervention specifically in children with DLD, with generally positive but methodologically limited findings. Pauls and Archibald [36] reported variable gains from narrative-based language intervention in a single-subject design with older children with DLD, noting that outcomes differed substantially across individuals and that a working memory impairment may attenuate response to intervention. Janssen et al. [37] demonstrated significant improvements in both narrative microstructure and macrostructure following a small-group narrative intervention for Dutch-speaking children with DLD; however, that study involved only six children per condition and did not include a business-as-usual control or follow-up assessment. More recently, Ibrahim et al. [38] conducted a pilot RCT of a novel narrative intervention program for Arabic-speaking children with DLD, demonstrating significant gains relative to conventional language training.

In a large-scale randomized controlled trial, Petersen et al. [39] reported that kindergarten students identified as at risk for language difficulty who received the small-group Story Champs [40] intervention significantly outperformed control peers on narrative retells and personal story generation. In some cases, students receiving intervention surpassed peers with advanced language abilities on narrative measures. Moreover, post-intervention differences between typically developing students and those identified as at risk were no longer evident for personal story generation, suggesting that explicit narrative intervention can meaningfully reduce language-based disparities.

These studies provide compelling evidence that explicit narrative language intervention improves narrative outcomes for children with and without language disorders. However, much of this work has focused on narrative outcomes in isolation, with less attention to how narrative intervention supports broader academic language expectations, including expository discourse.

## 4. Expository Language: Development and Intervention

Expository language places distinct and often greater demands on children, requiring the integration of unfamiliar content, abstract vocabulary, and less predictable discourse structures [41,42]. These demands become increasingly prominent as students move through the elementary grades [43,44]. For many students, difficulties with academic language emerge most clearly when they encounter expository discourse rather than narrative text, or when demands shift from narrative to informational language [44]. Understanding context, vocabulary, morphology, and inferential relationships all contribute to children’s ability to comprehend and generate expository language [14,45,46].

Unlike narratives, which are organized around causal and goal-directed episodes, expository texts are structured to convey information. They may be organized descriptively, sequentially, or comparatively, each requiring different linguistic resources and comprehension strategies. Gillam et al. [45] suggested that expository discourse is often more challenging for children to understand, recall, and generate because it lacks the causal structure that supports comprehension in narratives. In their study of fourth-grade students, 90% of children with typical language produced accurate paraphrases of expository passages during think-aloud tasks, compared to only 65% of peers with language impairment. Notably, paraphrase accuracy strongly predicted performance on comprehension questions, underscoring the close relationship between expressive expository language and comprehension. For children with DLD, these difficulties are compounded by the vocabulary, syntactic, and inferential demands that characterize expository texts, the very areas in which this population demonstrates the most persistent weaknesses [47,48].

Emerging evidence indicates that oral language intervention can support expository language development, though findings vary by instructional focus. Petersen et al. [39] found that kindergarten students receiving oral narrative language intervention showed significant gains in expository retell ability, with at-risk students improving to the point that their performance no longer differed from that of typically developing peers. These findings suggest that strengthening narrative language may benefit expository discourse, potentially through shared underlying language processes such as vocabulary, syntax, and discourse organization. However, other research indicates that direct instruction in expository language may be necessary to optimize outcomes [45]. Ukrainetz [49] demonstrated that students with language-related learning disabilities who received explicit, strategy-based expository instruction significantly outperformed control peers on expository note-taking and oral reporting, pointing to the value of targeted expository language instruction as a distinct intervention focus. These findings raise an important and unresolved question: To what extent can narrative- and expository-based language instruction support narrative and expository language development, and how much explicit expository instruction is required to produce meaningful gains?

Addressing this question is particularly important for children with DLD, who often experience persistent difficulties with both narrative and expository discourse [17]. Research is needed to examine the effects of integrated oral narrative and expository language intervention on both discourse genres. The present study addresses this gap by evaluating the impact of a dual-narrative and expository oral language intervention on narrative and expository language outcomes in children with DLD.

Despite the centrality of narrative and expository discourse in academic contexts, explicit and comprehensive oral language instruction remains limited in many early elementary classrooms. While whole-class instruction may be sufficient for some students, children with DLD often require more explicit, structured, and intensive instruction delivered in small-group settings to achieve meaningful gains. Instructional approaches that integrate narrative and expository language, emphasize active responding, and systematically scaffold linguistic complexity are especially well suited to meet these needs [29]. Ukrainetz [8] emphasized the need for manualized language curricula that provide intensive, interactive language experiences across instructional contexts, an approach that directly informs the intervention examined in the present study.

## 5. Purpose

Children with DLD are at elevated risk for persistent academic difficulties because they often require explicit, systematic oral language instruction delivered at sufficient intensity to support meaningful language growth. Narrative and expository discourse are foundational forms of academic language that support comprehension, learning, and later literacy development; however, relatively little experimental research has examined the effects of structured oral language intervention targeting both discourse genres concurrently, particularly for children with DLD. Moreover, maintenance of treatment effects beyond the immediate post-intervention period has rarely been examined in randomized controlled trials of oral language interventions.

The purpose of this study was to examine the effects of a structured, small-group oral language intervention targeting both narrative and expository discourse on oral language outcomes for early elementary-age students. Using a randomized controlled design, students were assigned to either an intervention condition or a business-as-usual control condition. Although the primary focus of the study was on children with DLD, the sample also included children with typical language, allowing for examination of whether language status moderated response to intervention. Outcomes were evaluated across three domains: (a) narrative language performance immediately following intervention, (b) expository language performance immediately following intervention, and (c) maintenance of narrative language gains two months after the conclusion of intervention.

By examining both immediate and maintenance effects of combined narrative and expository oral language intervention, and by evaluating differential response as a function of language status, this study aims to inform instructional decision-making for children with DLD and to clarify the role of explicit oral language intervention in supporting academic language development for early elementary grade students with and without DLD.

## 6. Research Questions

This randomized controlled trial examined the effects of a structured, small-group oral language intervention targeting narrative and expository discourse on language outcomes for elementary-aged students with and without DLD. Specifically, the study addressed the following research questions:

RQ1 (Primary Efficacy—Narrative Language): What is the effect of the intervention, compared to business-as-usual instruction, on students’ narrative language performance immediately following intervention, after controlling for baseline narrative language ability?

RQ2 (Moderation by Language Status): Does language status (DLD vs. typical language) moderate the effect of the intervention on post-intervention narrative language outcomes?

RQ3 (Subgroup Effects—DLD): What is the effect of the intervention on narrative language outcomes for students with DLD?

RQ4 (Immediate Effects—Expository Language): What is the effect of the intervention, compared to business-as-usual instruction, on students’ expository language comprehension and expression immediately following intervention?

RQ5 (Maintenance of Narrative Language Gains): To what extent are treatment-related gains in narrative language maintained two months after the conclusion of intervention?

## 7. Method

### Participants

Approval to conduct this study was obtained from a University Institutional Review Board. Participants were drawn from a pool of 394 kindergarten through second-grade students enrolled in three elementary schools across three school districts. All students were screened to identify those with DLD for study eligibility. Kindergarten students were assessed using the Predictive Early Assessment of Reading and Language (PEARL; [50]) and first and second grade students were assessed using the Dynamic Measures of Narrative Language and Decoding (DYMOND; [51]). The DYMOND is an individually administered, norm-referenced dynamic assessment designed to identify language learning disorder and dyslexia. Using a test-teach-test format, the DYMOND measures both current performance and learning potential, yielding sensitivity and specificity at or above 90%, across diverse cultural, linguistic, and socioeconomic groups. The PEARL [50] is a PreK and kindergarten criterion-referenced dynamic assessment screener designed to identify children at risk for future language and decoding learning difficulties before formal reading instruction begins. Like the DYMOND, the PEARL was developed to minimize cultural and linguistic bias, with sensitivity and specificity at or above 80% across diverse student populations. Students were classified as having DLD consistent with published decision criteria in the PEARL and DYMOND manuals.

Of the 394 students, 52 were identified as having DLD (13%), and 28 were identified as having typical language learning ability yet significantly weak language performance, where they could not produce a minimally complete narrative episode (a pretest total score of 9 or lower). Students were individually randomized to either a treatment or control condition. Narrative and expository language outcomes were collected for all randomized participants. There was no missing data for any administered outcome. Descriptive statistics for the final sample are presented in Table 1.

## 8. Measures

### 8.1. Narrative Language Measures (NLM)

Students’ narrative language abilities were assessed using the Narrative Language Measures (NLM) from the CUBED-3 assessment [52]. The NLM is a narrative language sample analysis approach that is a criterion-referenced, general outcome measure designed for children from preschool through eighth grade and is commonly used for diagnostic, screening, and progress-monitoring purposes. The assessment consists of 25 parallel forms per grade level, allowing for repeated administration with minimal practice effects. Administration typically requires approximately 3–5 min per student.

In the present study, one grade-appropriate NLM form was administered at pretest and a different form was administered immediately following the four-month narrative intervention as a primary outcome measure of oral narrative language. To examine maintenance of treatment effects, a third NLM parallel form was administered again two months after the intervention concluded. During the intervention phase, the NLM was additionally used as a progress-monitoring measure for students in both at-risk and not-at-risk language intervention groups. Means and standard deviations of the NLM pretest, posttest, and maintenance are displayed in Table 2.

Administration followed standardized procedures. Examiners read a brief, personally themed narrative aloud to the student without using visual supports or scaffolding. Students were instructed to listen carefully and then retell the story to the best of their ability. During retells, examiners provided only neutral prompts (e.g., “Keep going”), and no corrective feedback or leading questions were used. No pictures or story maps were provided in order to reflect students’ independent comprehension and expressive narrative abilities.

Following the retell, students answered a small set of story-related questions, including factual comprehension, inferential vocabulary, and inferential reasoning questions related to the narrative. These questions provided additional information about students’ understanding of the story content and their ability to make inferences beyond recall alone.

Narrative retells were scored in real time using the NLM scoring rubric, which captures multiple dimensions of narrative language performance. Points were awarded for the presence and completeness of story grammar elements, including character, setting, problem, feeling, action, consequence, and ending. Each element was scored on a 0–2 scale, with additional weighting for episodic structure (i.e., coordinated inclusion of problem, action, and consequence). Episodic complexity scores reflected the extent to which story grammar elements were integrated into coherent episodes rather than listed in isolation. In addition to discourse structure, the NLM captures selected features of sentence complexity. These include the use of temporal and causal subordinating conjunctions (e.g., after, when, because) and relative clauses. Vocabulary complexity was scored based on the frequency of use of tier-2 words. Responses to inferential vocabulary questions and inferential reasoning questions, where students are asked to make inferences based on information from the story and from their life experiences were scored. A total narrative retell score was computed by summing discourse structure, episodic complexity, sentence complexity, vocabulary complexity, factual questions, inferential vocabulary, and inferential reasoning. This composite score served as the primary narrative outcome variable for analyses.

The NLM has demonstrated good to excellent reliability and validity across multiple studies. Petersen and Spencer [50,53] reported strong interrater reliability, internal consistency, and concurrent criterion-related validity. Additional psychometric evidence, including predictive validity, sensitivity and specificity, and benchmark development, has been reported for over 4000 students in preschool through third grade in the CUBED Technical Manual [50]. Grade-level benchmark expectations were derived using regression-based methods aligned with state curriculum standards. In the present study, grade-appropriate benchmarks were used to contextualize student performance but were not used as cutoff scores for eligibility or grouping. The NLM has also been used as a primary outcome measure by researchers independent of the assessment’s authors, including Risueño et al. [54], who employed the NLM in a peer-reviewed study with bilingual students with DLD.

### 8.2. Expository Language

Students’ expository language comprehension and expression were assessed immediately after the intervention using a researcher-developed expository retell task designed to measure students’ ability to comprehend, retain, and express unfamiliar informational content. The expository passage used in the present study was the Sea Pig passage, an informational text describing an unfamiliar deep-sea creature (*Scotoplanes globosa*) and its characteristics, habitat, and behaviors. The passage was selected because its content is highly unlikely to be familiar to young children, ensuring that retell performance reflected comprehension and expressive language ability rather than prior knowledge. The Sea Pig passage has been used as the primary expository outcome measure in prior large-scale research [39] and follows the same general administration and scoring logic as the NLM.

During administration, the examiner orally presented a brief informational passage. The passage was read aloud slowly, following standardized examiner scripts and pacing procedures. To reduce working memory demands and better isolate comprehension and expressive language abilities, students were permitted to take notes or draw while listening. After the presentation, students were asked to retell the information they remembered from the passage. Retells were scored in real time using a structured rubric that captured the accuracy and completeness of students’ expression of the passage’s main idea and supporting details. Scoring differentiated between accurate production of key informational units, partial or imprecise responses, and omissions (0–2). This approach allowed for the sensitive measurement of students’ ability to organize and verbally express newly learned information.

After the retell, students answered a set of comprehension questions about the passage. These included factual text-based questions as well as inferential vocabulary questions targeting key academic words embedded in the passage (e.g., unusual, dwells, rotten). Vocabulary understanding was assessed using a combination of definition and context-based questions, with follow-up prompts provided when initial responses were incorrect. This design allowed examiners to evaluate students’ ability to infer word meaning from linguistic context rather than relying on prior lexical knowledge.

In addition to information recall and comprehension, the assessment included a brief analysis of students’ use of selected language structures (e.g., causal connectives, relative clauses, and target academic vocabulary) during retells and responses. Credit was awarded when these forms were used spontaneously and meaningfully, providing an index of students’ expressive language complexity in the context of expository discourse.

Expository language outcomes were assessed at posttest only. Unlike narrative language, expository language was not assessed at pretest or at the two-month maintenance time point. This decision was driven by practical constraints inherent to school-based data collection: participating schools imposed strict limitations on the total assessment time permissible across the study period, and available testing time was allocated first to the primary narrative language outcome measures, which were assessed at pretest, posttest, and maintenance.

### 8.3. Treatment Condition: Story Champs Small-Group Intervention

Students assigned to the treatment condition received small-group language intervention twice weekly for approximately 15–20 min per session over an eight-week period, following the Story Champs explicit and systematic instructional framework [40]. Intervention was delivered by trained undergraduate and graduate students. Groups consisted of three to four students identified as having emerging academic language skills or language disorder based on dynamic assessment results. Instruction alternated between narrative and expository language activities across sessions.

### 8.4. Narrative Language Intervention

Narrative instruction targeted students’ understanding and production of story structure using personally themed narratives. For each session, the interventionist selected a story from the Story Champs storybook and corresponding illustrations, story grammar icons, and a structured participation game. Instruction began with explicit modeling, during which the interventionist read the story aloud while displaying illustrations and placing story grammar icons (e.g., character, problem, action, consequence, ending) near the corresponding story events. Students were taught to identify and label each story grammar component as it occurred. Following modeling, students participated in a supported team retell, in which each student was assigned a story grammar icon and retold their portion of the story in sequence. The interventionist provided scaffolding as needed and summarized the complete narrative to ensure all elements were included. Instruction then progressed to individual narrative retells, first with full visual supports (illustrations and icons), then with reduced supports (icons only), and finally without visual supports. Sessions concluded with personal story generation, during which students produced their own narratives using the same story grammar framework. Visual supports were systematically faded across personal storytelling to promote independent narrative organization. These procedures are described in detail in the Story Champs manual and prior studies [39,40].

### 8.5. Expository Language Intervention

Expository instruction targeted students’ ability to comprehend, organize, and retell informational text. Interventionists selected grade-level informational passages (approximately 65–130 words) and reorganized the content into a structured pattern block consisting of a main idea and four key supporting details. Sessions began with explicit modeling, during which the interventionist read the passage aloud while placing main idea and supporting detail icons on the pattern block. Students were taught to take brief notes using words or drawings aligned with each informational unit. Early sessions emphasized simplified structures (sentence-level main idea with single-word details), with gradual progression to sentence-level expression for all components. Instructions included explicit vocabulary instruction for unfamiliar terms identified within the passage. New terms were introduced using a consistent routine that involved definition, repetition, and contextualized use. Students then participated in a team retell, with each student responsible for retelling a portion of the passage using icons and notes as supports. The interventionist modeled accurate responses and facilitated group repetition to reinforce complete and precise language. Instruction progressed to individual expository retells, with systematic fading of supports across successive retell attempts, from icons and notes to notes only.

All intervention procedures adhered to standardized lesson plans and fidelity guidelines outlined in the Story Champs manual, which provided step-by-step procedures for each instructional session. Interventionists were required to demonstrate 100% procedural fidelity during an initial training phase supervised by a certified speech–language pathologist before delivering any sessions with students. Following training, the on-site supervisor observed each interventionist on a weekly basis and provided structured feedback to ensure continued adherence to instructional sequences and support-fading procedures. To ensure examiner objectivity, all examiners who administered and scored assessments were blinded to the treatment condition. Students were assigned random identification numbers prior to data collection, and examiners were not informed of group assignment. The treatment condition was known only to the principal investigator, who was not involved in assessment scoring.

### 8.6. Business as Usual Control Group

A business-as-usual control condition was selected because it reflects the real-world counterfactual of greatest relevance to practitioners and administrators: whether structured small-group oral language intervention produces outcomes beyond what children would achieve through typical school instruction. Withholding intervention from an at-risk population is ethically justifiable only when access to that intervention is available following the study period. Consistent with this ethical commitment, all students assigned to the control condition were offered the Story Champs intervention during the following school year.

All teachers in the participating schools were receiving training in Language Essentials for Teachers of Reading and Spelling (LETRS^®^). LETRS^®^ is a comprehensive professional learning program providing elementary school educators and administrators with in-depth knowledge to become experts in the science of reading. LETRS^®^ was developed to teach educators the fundamental skills necessary to teach literacy [55]. Additionally, the schools were implementing 95% Group reading intervention. This program was developed by the 95 Percent Group, which has a mission to raise the percentage of students reading at grade level to ninety-five percent. These programs are customizable based on the specific needs observed in various school districts, and available kits include teacher lesson plans and student workbooks to facilitate literacy acquisition and bridge the gap between students’ current reading levels and those expected for their grade (95 Percent Group, n.d.) [56]. Importantly, neither LETRS professional development nor 95% Group reading intervention provide explicit and systematic language instruction procedures in the way the Story Champs intervention does. As such, students in the control condition received substantive literacy instruction with a focus on word recognition but were not exposed to the explicit oral language instruction that defined the treatment condition.

## 9. Results

### 9.1. Preliminary Analyses

Descriptive statistics for all outcome variables by condition are presented in Table 2. Independent-samples *t* tests indicated no statistically significant differences between the intervention and control groups at baseline on narrative language performance, *t*(78) = 1.58, *p* = 0.12, suggesting that random assignment produced fairly comparable groups prior to the intervention.

Assumptions for ANCOVA and regression analyses were evaluated prior to hypothesis testing. Visual inspection of residual plots supported assumptions of normality, linearity, and homoscedasticity. The assumption of homogeneity of regression slopes was tested by examining the interaction between treatment condition and pretest narrative language. The interaction was not statistically significant, *F*(1, 76) = 2.59, *p* = 0.11, indicating that the relationship between the covariate and the outcome did not differ across treatment groups. Levene’s test indicated that the assumption of homogeneity of variance was met for narrative language outcomes (*p* > 0.05). For the expository language outcome, regression assumptions were evaluated through inspection of residual plots and influence statistics. No violations of linearity, homoscedasticity, or undue influence were observed. There were no missing data for narrative or expository outcomes.

### 9.2. Research Question 1: Immediate Effects on Narrative Language

To examine the immediate effects of the intervention on narrative language outcomes, an analysis of covariance (ANCOVA) was conducted with post-intervention narrative language scores as the dependent variable, treatment condition (intervention vs. control) as the fixed factor, and pretest narrative language performance as the covariate.

After adjusting for baseline narrative language ability, there was a statistically significant main effect of treatment condition on post-intervention narrative language performance, F(1, 77) = 13.67, *p* < 0.001, partial η^2^ = 0.15. Adjusted mean narrative language scores were higher for students in the intervention group (Madj = 10.34, SE = 0.81) than for students in the control group (Madj = 6.06, SE = 0.81). The adjusted mean difference between groups was 4.28 points (95% CI 1.97, 6.58). These findings indicate that participation in the structured small-group oral language intervention resulted in significantly greater improvements in narrative language performance compared to business-as-usual instruction.

### 9.3. Research Question 2: Moderation by Language Status (DLD vs. Typical Language)

To evaluate whether intervention effects differed as a function of language status, a second ANCOVA model was estimated that included treatment condition, language status (DLD vs. typical language), and their interaction, with pretest narrative language performance included as a covariate.

Results revealed no statistically significant Treatment × Language Status interaction, F(1, 75) = 0.32, *p* = 0.58, partial η^2^ = 0.01, indicating that the magnitude of the intervention effect did not differ between students with DLD and those with typical language. Thus, the intervention was similarly effective for students with and without DLD.

### 9.4. Research Question 3: Subgroup Effects for Students with DLD

Although the Treatment × Language Status interaction was not statistically significant, we conducted a planned subgroup analysis to characterize intervention effects specifically for students with DLD, given the clinical relevance of this population. Thus, to further characterize intervention effects for students with DLD, an ANCOVA was conducted within the DLD subgroup only (n = 52), with post-intervention narrative language scores as the dependent variable, treatment condition as the fixed factor, and pretest narrative language performance as the covariate. After adjusting for baseline performance, students with DLD who received the intervention demonstrated significantly higher post-intervention narrative language scores than students with DLD in the control condition, F(1, 49) = 18.89, *p* < 0.001, partial η^2^ = 0.28. The adjusted mean difference was 5.10 points (95% CI 2.74, 7.46), representing a large effect. These results indicate that the intervention was effective for students with DLD.

### 9.5. Research Question 4: Immediate Effects on Expository Language

To evaluate intervention effects on expository language outcomes, linear regression analyses were conducted using post-intervention expository language scores as the dependent variable and treatment condition as the primary predictor. Because an expository language pretest was not administered, intervention effects on expository outcomes were estimated using linear regression models with treatment condition as the primary predictor and pretest narrative language included as a covariate to adjust for baseline oral language ability.

The overall model was statistically significant, F(2, 77) = 13.99, *p* < 0.001, accounting for 26.7% of the variance in post-intervention expository language performance (R^2^ = 0.27, adjusted R^2^ = 0.25). Treatment condition was not a statistically significant predictor of the expository language outcome after controlling for pretest narrative language ability, B = 2.55, SE = 1.55, β = 0.16, t(77) = 1.65, *p* = 0.10, 95% CI −0.54, 5.64. Students in the treatment group scored on average 2.55 points higher on the expository posttest than students in the control condition after adjusting for baseline oral language ability. The NLM pretest covariate was a statistically significant predictor (B = 1.21, SE = 0.26, β = 0.46, t(77) = 4.66, *p* < 0.001, 95% CI 0.69, 1.73), indicating that baseline oral language ability was positively associated with expository language performance at posttest. These findings indicate a positive trend favoring the treatment group on expository language outcomes; however, this difference did not reach statistical significance after adjusting for baseline oral narrative language ability.

### 9.6. Research Question 5: Maintenance of Narrative Language Gains

To examine maintenance of narrative language gains, a follow-up ANCOVA was conducted, with narrative language scores collected two months after the intervention as the dependent variable, treatment condition as the fixed factor, and pretest narrative language performance as the covariate. Results indicated a statistically significant effect of treatment condition at follow-up, F(1, 77) = 36.27, *p* = < 0.001, partial η^2^ = 0.30. Students in the intervention group maintained higher narrative language performance at follow-up (Madj = 15.31, SE = 1.00) compared to students in the control group (Madj = 7.18, SE = 1.00).

## 10. Discussion

Guided by the view of oral academic language as a foundational system supporting comprehension, learning, and later literacy development, these findings directly address the gaps identified in the introduction regarding the need for explicit instruction, broader genre coverage, and evidence of durability. The purpose of this study was to examine the effects of a structured small-group oral language intervention targeting both narrative and expository discourse on academic language outcomes for early elementary-age students. Narrative and expository discourse represent core forms of academic language that support comprehension, learning, and later literacy development, yet they are rarely addressed together within a single, systematic instructional approach. Across analyses of this randomized controlled design, the structured small-group oral language intervention produced significant and meaningful improvements in narrative language outcomes compared with business-as-usual instruction. Treatment effects were observed across the full sample, were not moderated by language status, and were robust among students with DLD. These gains were maintained two months following the conclusion of the intervention, suggesting that the effects were durable rather than transient. Expository language outcomes showed a positive trend favoring the treatment group but did not reach statistical significance, likely reflecting the absence of a direct expository baseline measure and the modest statistical power available for that analysis. These findings provide evidence that explicit, structured oral language intervention can yield meaningful and lasting improvements in narrative language for early elementary-age students, including those with developmental language disorder, while also suggesting that expository language gains may require greater instructional intensity, longer intervention duration, or more direct expository language targets to produce reliable and detectable effects.

These findings extend prior intervention research with children with DLD and children who would benefit from tier-2 language intervention. Unlike Pauls and Archibald [36], who reported variable and mixed individual outcomes using a single-subject clinical model, the present randomized controlled design demonstrated consistent group-level effects favoring the intervention, with large effects specifically for the DLD subgroup. The present findings also extend Janssen et al. [37], who showed that small-group narrative intervention can improve narrative macro- and microstructure in children with DLD, by demonstrating these effects in a larger, randomized sample, within an authentic school-based Tier 2 service delivery model, and with maintenance of gains confirmed two months following the intervention. The present study adds to the growing international evidence base for narrative intervention in DLD, including recent RCT evidence from Arabic-speaking populations [38], by providing experimental evidence from a manualized English-language program that concurrently targets both narrative and expository discourse.

These findings extend prior work demonstrating that oral language skills are responsive to explicit instruction [27,28,39] in several important ways. First, the present study provides experimental evidence that instruction simultaneously targeting narrative and expository discourse can produce meaningful gains in academic language. Second, it demonstrates that these gains are not transient; rather, narrative language improvements were maintained beyond the immediate intervention period. Third, the results indicate that structured oral language instruction is effective for students with DLD as well as for peers with typical language, supporting its relevance across a continuum of language ability. These findings address key gaps identified in the literature regarding the durability, generalization, and instructional scope of oral language intervention in the early grades.

Throughout the intervention, students were repeatedly engaged in listening to structured narratives and informational passages, retelling content multiple times, and producing increasingly complete and complex oral language with systematic scaffolding and prompt fading. This emphasis on active language production stands in contrast to more passive language exposure models and reflects a deliberate focus on helping students construct and externalize coherent mental representations of content. It is theoretically plausible that this emphasis supported the integration of vocabulary, syntax, and discourse-level organization—the processes most central to academic language development—though the present study was not designed to isolate or verify these mechanisms directly.

Explicit and repeated attention to Tier 2 vocabulary across both narrative and expository activities likely supported students’ ability to represent and express key ideas with greater precision. However, because story grammar elements and vocabulary complexity are both components of the NLM composite score that served as the primary outcome, it is not possible to determine from the present analyses whether gains in story grammar structure, vocabulary, sentence complexity, or their interaction drove overall improvement. The close alignment between instructional emphasis and observed outcome gains is encouraging and consistent with a theoretically motivated account of change, but formal mediation testing would be required to substantiate these mechanisms. Future research incorporating independent measures of vocabulary, syntax, and discourse structure, assessed separately from the outcome composite, would enable more rigorous examination of the processes through which structured oral language instruction produces its effects.

### 10.1. Narrative Language Outcomes and Instructional Mechanisms

The significant gains observed in narrative language performance are consistent with a robust body of prior research demonstrating that narrative discourse is highly responsive to explicit instruction [27,28,33,39]. Narrative discourse serves as a critical bridge between oral and written language, requiring children to coordinate story grammar, language complexity, and episodic structure. The intervention examined in this study directly targeted these components through repeated opportunities to listen to, retell, and generate narratives with systematic scaffolding of story grammar elements and linguistic complexity.

This instructional approach aligns with theoretical models positing that narrative structure provides a cognitively efficient framework for organizing linguistic information and supports integration across multiple levels of language. By repeatedly engaging students in structured narrative production and gradually reducing instructional supports, the intervention likely facilitated the development of more coherent, complete, and independently produced narrative representations. Importantly, this approach emphasizes learning how to organize language, not merely recalling story content, which may help explain the durability of gains observed at follow-up.

Notably, the magnitude of narrative language gains observed in this study suggests that even relatively brief, focused oral language instruction can yield meaningful improvements when instruction is explicit, cumulative, and aligned with core language constructs. This finding is particularly important given the instructional constraints faced by schools and clinicians. Moreover, the results extend prior work by demonstrating that narrative language intervention can be effective not only for students with emerging language weaknesses but also for students with more persistent language impairments, including those with DLD. In doing so, the present study strengthens the evidence base supporting structured narrative intervention as a viable and impactful instructional approach for diverse learners in the early elementary grades. These findings are consistent with the Simple View of Reading framework insofar as they demonstrate that the language comprehension component of reading, specifically at the discourse level, is responsive to explicit instruction. However, the present study did not measure decoding or reading comprehension directly, and future research is needed to examine whether gains in oral narrative language translate to improvements in reading comprehension outcomes.

### 10.2. Expository Language Outcomes and Instructional Mechanisms

Expository discourse places distinct linguistic demands on learners and is often both developmentally underestimated and instructionally neglected in the early grades, particularly for students with DLD. Expository language was intentionally and systematically targeted alongside narrative discourse throughout the intervention; however, the intervention did not produce a statistically significant effect on expository language outcomes after adjusting for baseline oral language ability. Students in the treatment condition scored on average 2.55 points higher on the expository posttest than control peers, a positive trend that did not reach significance. These findings should be interpreted with caution given the absence of a direct expository pretest, the use of narrative language as a proxy covariate, and the resulting limitations on statistical precision.

Although expository gains did not reach statistical significance, the pattern of results is noteworthy given the well-documented challenges associated with expository discourse. Expository language differs fundamentally from narrative language in structure, purpose, and linguistic demands. Informational texts often lack the causal and temporal coherence that characterizes narratives, requiring students to rely more heavily on vocabulary knowledge, syntactic precision, and discourse organization to extract and convey meaning [45,46]. For many children, particularly those with language impairments, these demands make expository discourse substantially more difficult to comprehend and produce. The present results add to a growing body of evidence suggesting that these challenges are not immutable, but rather responsive to targeted instruction.

The expository component of the intervention emphasized identifying main ideas, organizing supporting details, and using explicit linguistic markers to convey informational content. Students were supported in constructing organized representations of informational passages through repeated listening, guided retelling, and gradual removal of scaffolds. This approach is consistent with prior work demonstrating that expository language skills improve when children are explicitly taught how information is structured and how ideas relate within a text [45]. The present study extends this literature by demonstrating that expository instruction can be successfully integrated with narrative instruction within a single intervention framework, rather than treated as a separate or later-developing instructional target.

Although statistically significant gains in expository language were not demonstrated, the positive trend observed in the present study is noteworthy given the relatively young age of participants. Expository discourse is often assumed to be developmentally inappropriate or instructionally premature for early elementary students. However, the present findings challenge this assumption [4]. When instruction is carefully scaffolded and linguistically accessible, young students are capable of engaging meaningfully with expository content. For students with DLD, this early exposure may be particularly important, as delays in expository language development can compound academic difficulties as informational texts become increasingly dominant in later grades.

These findings are consistent with the inclusion of expository discourse as a core component of early oral language intervention rather than an instructional add-on reserved for later grades, though further research is needed to confirm whether this intervention approach reliably produces detectable expository gains. By addressing both narrative and expository language concurrently, the intervention reflects the reality of classroom language demands and provides students with tools needed to navigate multiple discourse genres. The findings suggest that early, explicit instruction in expository language is both feasible and may be beneficial, even for students with significant language learning challenges.

### 10.3. Maintenance of Narrative Language Gains

The maintenance of narrative language gains two months following intervention provides compelling evidence that observed improvements were not transient. Students in the intervention group continued to outperform their peers receiving business-as-usual instruction at follow-up, despite the absence of ongoing treatment. This pattern suggests that the intervention supported durable changes in students’ narrative language abilities rather than short-lived performance effects attributable to repeated practice or proximity to instruction.

From a theoretical perspective, maintenance effects are consistent with models of language learning that emphasize internalization of linguistic structures through repeated, scaffolded practice. Throughout the intervention, students were provided with systematic opportunities to analyze, organize, and produce narratives with gradually reduced instructional support. This instructional sequence likely facilitated the development of stable narrative schemas and language routines that students could access independently once explicit scaffolding was removed. In this sense, maintenance reflects not merely the retention of content but the consolidation of underlying discourse-level competencies.

Maintenance findings are particularly salient for students with DLD, for whom intervention effects often attenuate when instructional support ends. Many language interventions demonstrate immediate post-treatment gains but provide limited evidence of long-term persistence. The sustained narrative language advantages observed in the present study suggest that explicit, structured oral language intervention may alter students’ developmental trajectories by strengthening foundational discourse skills that continue to support language production beyond the intervention window. This durability is especially important in early elementary grades, where language demands rapidly increase, and early weaknesses can compound if left unaddressed. By demonstrating maintenance of narrative language gains beyond the immediate intervention period, this study directly addresses a limitation identified in prior oral language intervention research, specifically, the lack of evidence for durability of effects [36].

### 10.4. Implications for Instruction and Practice

The findings from this study have several important implications for educational practice. First, they provide strong support for the inclusion of explicit oral language instruction within early elementary curricula, particularly for students with identified language vulnerabilities. Narrative and expository discourse are not ancillary skills; they represent core forms of academic language through which students are expected to comprehend instruction, demonstrate knowledge, and engage in learning. Targeted instruction in these domains may therefore play a critical role in preventing downstream difficulties in comprehension and academic achievement.

Second, the results underscore the feasibility and effectiveness of delivering structured oral language intervention in small-group settings. Small-group instruction allowed for high rates of active responding, individualized scaffolding, and immediate feedback, features especially important for students with DLD. Within multi-tiered systems of support, such approaches may serve as an efficient Tier 2 option for students who require more intensive language instruction than can be provided through whole-class teaching alone.

Importantly, the intervention examined in this study required a relatively modest instructional investment. Despite its limited duration, the intervention produced meaningful and sustained gains in academic language. This finding has practical significance for schools and clinicians navigating constraints related to time, staffing, and competing instructional priorities. It suggests that well-designed, linguistically focused interventions can yield substantial benefits without imposing unrealistic demands on instructional schedules.

More broadly, these findings highlight the value of shifting from reactive models of language intervention, implemented only after academic failure becomes evident, to proactive approaches that strengthen academic language early. Embedding explicit narrative and expository language instruction within early intervention frameworks may help reduce the cumulative impact of language weaknesses and support more equitable learning outcomes for students with and without language disorders.

### 10.5. Effects for Students with and Without DLD

An important contribution of the present study is the demonstration that structured oral language intervention benefited students across a continuum of language ability. Although students with DLD entered the study with lower baseline narrative language performance, intervention effects were not moderated by language status. That is, students with DLD and those with typical language both demonstrated significant gains in narrative language following intervention, and the intervention’s relative advantage did not differ as a function of diagnostic classification.

This finding aligns with prior work suggesting that explicit, systematic language instruction supports learning for all students, particularly for those with language vulnerabilities. From an instructional perspective, this pattern reinforces the value of designing language interventions that are universally effective yet sufficiently explicit and scaffolded to meet the needs of students with more persistent language impairments. Rather than requiring fundamentally different instructional approaches for students with and without DLD, the results suggest that high-quality language instruction grounded in core discourse structures can support growth across learner profiles.

Although the Treatment × Language Status interaction was not statistically significant, this should be interpreted as an absence of evidence for differential responsiveness rather than evidence that students with DLD and typical language benefit identically. Moderation analyses are often underpowered, and the present study was designed primarily to test overall efficacy. Importantly, subgroup analyses demonstrated that students with DLD showed large, meaningful gains relative to peers with DLD receiving business-as-usual instruction, indicating that the intervention is not only broadly effective but also clinically relevant for children with persistent language learning needs. This finding is especially noteworthy given the well-documented persistence of language difficulties for students with DLD and the limited responsiveness often observed in less explicit instructional contexts. The present results suggest that when instruction directly targets narrative and expository discourse structures through repeated, supported language production, students with DLD can make substantial progress.

These findings support a prevention-oriented view of language intervention. Explicit oral language instruction need not be reserved solely for students with identified disabilities, nor should it be delayed until academic difficulties become entrenched. Instead, the same instructional principles that benefit students with DLD may also strengthen academic language for typically developing peers, potentially narrowing performance gaps and supporting more inclusive instructional models.

The present study extends existing research on oral language intervention in several important ways. Prior investigations have demonstrated that narrative language skills can improve following explicit instruction, but much of this work has relied on single discourse genres, immediate post-treatment outcomes only, or non-randomized designs. The current randomized controlled trial examined the effects of a structured intervention targeting both narrative and expository discourse, included students with and without DLD, and assessed maintenance of narrative language gains beyond the intervention period. The findings also build on prior Story Champs research by demonstrating that its core instructional principles of explicit modeling, repeated retelling, systematic scaffolding, and prompt fading, can be applied effectively across discourse genres and learner profiles, supporting not only immediate performance gains but more enduring changes in language ability.

### 10.6. Limitations and Future Directions

Several limitations should be considered when interpreting the findings. First, expository language outcomes were assessed at posttest only, as school-imposed constraints on assessment time precluded expository data collection at pretest and at the two-month maintenance time point. The absence of a direct expository baseline measure necessitated the use of pretest narrative language performance as a covariate to adjust for baseline oral language ability in expository outcome analyses, which represents an imperfect but theoretically justified approach given the established relationship between narrative expository language competence. There was only a modest correlation between the narrative pretest and covariate and expository posttest scores (*r* = 0.49), suggesting that meaningful variance in expository outcomes remained unexplained, which may have attenuated the precision of the treatment effect estimate. Additionally, the absence of expository maintenance data precluded examination of whether any expository gains were sustained over time. Future research should incorporate direct expository language assessments at all time points to enable more rigorous and complete evaluation of intervention effects on expository outcomes. Future research should also examine whether greater instructional intensity, longer intervention duration, or more explicit expository language targets produce stronger and more detectable expository language gains.

Due to the limited sample size, analyses were not conducted to examine the effect of the intervention on the culturally and linguistically diverse students included in this study. Future research should explore any potential differential responses to the intervention.

Although interventionists demonstrated 100% procedural fidelity during the initial training phase and were observed weekly by the on-site supervisor throughout the intervention period, continuous fidelity data were not formally documented with quantitative indices across all sessions. In the context of a randomized controlled trial, ongoing quantitative fidelity monitoring is an important standard for ensuring that observed effects are attributable to the intervention rather than to undetected variation in delivery. The absence of session-by-session fidelity data therefore represents a limitation of the present study. Future research should incorporate systematic quantitative fidelity documentation across all phases of implementation using standardized checklists or behavioral observation protocols. Furthermore, the nature, frequency, and duration of any oral narrative or expository language instruction delivered in the control classrooms was not formally documented. Without this information, it is not possible to characterize the control condition adequately to rule out the possibility that some control students received incidental oral language instruction that partially overlapped with intervention targets. Future research should document control condition instruction in greater detail.

The present analyses establish that the intervention produced significant gains in narrative language outcomes but were not designed to explain the mechanisms of change. Although it is theoretically plausible that gains in story grammar structure, vocabulary, sentence complexity, or their interaction drove overall improvement, the NLM composite score, which served as the primary outcome, incorporates story grammar, vocabulary, sentence complexity, and comprehension as component scores. Using these components as mediators of their own composite would introduce circularity that would complicate interpretation. Furthermore, independent assessments of potential mediating variables, such as standardized vocabulary measures or discrete story grammar production tasks administered separately from the outcome measure, were not collected. Future research should incorporate such measures at multiple time points to enable formal mediation testing and provide evidence regarding the cognitive and linguistic processes through which structured oral language instruction produces its effects.

While maintenance of narrative language gains was demonstrated over a two-month period, longer-term follow-up is needed to determine whether improvements persist across subsequent academic years and translate into broader outcomes such as reading comprehension and written expression. Longitudinal studies examining how early oral language intervention influences later literacy trajectories would be especially valuable.

The intervention was delivered in small-group settings by trained interventionists. Future research should examine the effectiveness of similar instructional approaches when implemented by classroom teachers and within larger-scale instructional models, including Tier 1 and Tier 2 frameworks. Such work would further inform scalability and implementation feasibility in authentic school contexts.

Despite these limitations, the present study provides evidence that explicit, structured oral language intervention targeting both narrative and expository discourse can produce meaningful and durable gains for young students. Future research should continue to refine instructional models, examine optimal dosage and delivery formats, and explore how early language intervention can be integrated within comprehensive literacy frameworks.

## 11. Conclusions

This randomized controlled trial provides evidence that explicit, structured oral language intervention targeting both narrative and expository discourse can produce meaningful and durable improvements in academic language for early elementary-age students. By addressing two core discourse genres within a single instructional framework, the intervention reflects the linguistic demands students encounter in real classroom contexts and demonstrates that young learners, including those with DLD, can make substantial progress when language instruction is systematic, scaffolded, and focused on active language production. The maintenance of narrative language gains further suggests that such instruction may support lasting changes in students’ language organization and use. These findings underscore the importance of early, intentional oral language instruction and support its inclusion as a central component of educational and intervention efforts to strengthen academic language foundations for diverse learners.

## Figures and Tables

**Table 1:** Demographic Information for Treatment and Control Students.

	Treatment Group *n* (%)	Control Group *n* (%)
Number of Students	40 (50.0%)	40 (50.0%)
*Gender*		
Male	22 (55%)	25 (62.5%)
Female	18 (45%)	15 (37.5%)
*Ethnicity*		
White	27 (67.5%)	32 (80%)
Hispanic	12 (30%)	6 (15%)
Asian	1 (2.5%)	2 (5%)
*Grade Level*		
K	30 (75%)	33 (82.5%)
1	3 (7.5%)	3 (7.5%)
2	7 (17.5%)	4 (10%)
*IEP for Language*		
Yes	1 (2.5%)	4 (10%)
No	39 (97.5%)	36 (90%)
Language Disorder	26 (65%)	26 (65%)
*Home Language*		
Spanish	12 (30%)	6 (15%)
English only	28 (70%)	34 (85%)
*Free/Reduced Lunch*		
Yes	13 (33.3%)	13 (33.3%)
No	27 (66.6%)	27 (66.6%)

**Table 2:** Means, Standard Deviations, and Statistical Significance for Treatment and Control Groups for Pretest and Posttest Measures.

	Treatment Group (*n* = 40)	Control Group (*n* = 40)	Significance
Pretest NLM	4.10 (3.45)	3.05 (2.41)	*p* = 0.12
Posttest NLM (Total Sample)	M = 10.75, SD = 6.07 Adjusted M = 10.34, SE = 0.81	M = 5.65, SD = 5.02 Adjusted M = 6.06, SE = 0.81	*p* < 0.001 partial η^2^ = 0.15
Posttest NLM (DLD Sample)	M = 9.11, SD = 4.32 Adjusted M = 8.84, SE = 0.82	M = 3.46, SD = 4.22 Adjusted M = 3.74, SE = 0.82	*p* < 0.001 partial η^2^ = 0.28
Expository Posttest	M = 14.05, SD = 7.86 Adjusted M = 13.42, SE = 1.09	M = 10.23, SD = 7.50 Adjusted M = 10.86, SE = 1.09	*p* = 0.10 partial η^2^ = 0.03
Maintenance NLM	M = 15.75, SD = 7.74 Adjusted M = 15.31, SE = 1.00	M = 6.75, SD = 5.39 Adjusted M = 7.18, SE = 1.00	*p* < 0.001 partial η^2^ = 30

Note: NLM = Narrative Language Measures subtest from the CUBED-3; M = Mean, SD = Standard Deviation, SE = Standard Error.

## Data Availability

The raw data supporting the conclusions of this article will be made available by the authors on request.
